# Colo-pancreaticoduodenectomy for locally advanced colon carcinoma—feasibility in patients presenting with acute abdomen

**DOI:** 10.1186/s13017-021-00351-6

**Published:** 2021-02-27

**Authors:** Joe-Bin Chen, Shao-Ciao Luo, Chou-Chen Chen, Cheng-Chung Wu, Yun Yen, Chuan-Hsun Chang, Yun-An Chen, Fang-Ku P’eng

**Affiliations:** 1 Department of Surgery, Taichung Veterans General Hospital, Taichung, Taiwan; 2 Department of Environmental Engineering, Dayeh University, Changhwa, Taiwan; 3 Institute of Medicine, Chung Shan Medical University, Taichung, Taiwan; 4 Department of Surgery, Faculty of Medicine, National Yang-Ming University, Taipei, Taiwan; 5 Cancer Translational Research Center, Taipei Medical University, Taipei, Taiwan; 6 Department of Surgery, Cheng Hsin General Hospital, Taipei, Taiwan; 7 Department of Pathology, Taichung Veterans General Hospital, Taiwan Boulevard Sec. 4, No. 1650, Taichung, 40705 Taiwan

**Keywords:** Locally advanced colon carcinoma, Pancreaticoduodenectomy, Colectomy, Acute abdomen

## Abstract

**Background:**

En bloc right hemicolectomy plus pancreaticoduodenectomy (PD) is administered for locally advanced colon carcinoma that invades the duodenum and/or pancreatic head. This procedure may also be called colo-pancreaticoduodenectomy (cPD). Patients with such carcinomas may present with acute abdomen. Emergency PD often leads to high postoperative morbidity and mortality. Here, we aimed to evaluate the feasibility and outcomes of emergency cPD for patients with advanced colon carcinoma manifesting as acute abdomen.

**Methods:**

We retrospectively reviewed 4898 patients with colorectal cancer who underwent curative colectomy during the period from 1994 to 2018. Among them, 30 had locally advanced right colon cancer and had received cPD. Among them, surgery was performed in 11 patients in emergency conditions (bowel obstruction: 6, perforation: 3, tumor bleeding: 2). Selection criteria for emergency cPD were the following: (1) age ≤ 60 years, (2) body mass index < 35 kg/m^2^, (3) no poorly controlled comorbidities, and (4) perforation time ≤ 6 h. Three patients did not meet the above criteria and received non-emergency cPD after a life-saving diverting ileostomy, followed by cPD performed 3 months later. We analyzed these patients in terms of their clinicopathological characteristics, the early and long-term postoperative outcomes, and compared findings between emergency cPD group (e-group, *n* = 11) and non-emergency cPD group (non-e-group, *n* = 19). After cPD, staged pancreaticojejunostomy was performed in all e-group patients, and on 15 of 19 patients in the non-e-group.

**Results:**

The non-e-group was older and had a higher incidence of associated comorbidities, while other clinicopathological characteristics were similar between the two groups. None of the patients in the two groups succumbed from cPD. The postoperative complication rate was 63.6% in the e-group and 42.1% in the non-e-group (*p* = 0.449). The 5-year overall survival rate were 15.9% in the e-group and 52.6% in the non-e-group (*p* = 0.192).

**Conclusions:**

Emergency cPD is feasible in highly selected patients if performed by experienced surgeons. The early and long-term positive outcomes of emergency cPD are similar to those after non-emergency cPD in patients with acute abdominal conditions.

## Background

The prevalence of colorectal carcinoma is increasing worldwide [1]. In Taiwan, this cancer has ranked highest among new malignant cases, especially over the last two decades [2]. The only promising cure at early stages is radical colectomy, performed for R0 resection [3]. For locally advanced colorectal carcinoma, en bloc resection is required for the involved adjacent organ [4]. Multiorgan resection for colorectal carcinoma is often associated with higher postoperative morbidity and mortality [3–5].

When right colon cancer directly infiltrates the duodenum near the ampulla of Vater or the pancreatic head, radical resection consists of right hemicolectomy plus pancreaticoduodenectomy (PD) [5–25]. We have called this procedure colo-pancreaticoduodenectomy (cPD).

PD techniques were demonstrated first by Kausch in Germany and by Whipple in the USA in the early twentieth century. This complex procedure has remained basically unchanged for nearly a century. The PD procedure, also known as the “Kausch-Whipple procedure” [26], is the standard for treating neoplasms or complex injuries or diseases involving the duodenum and pancreatic head [26–34]. Recently, despite lowering of the operative mortality of PD to <5%, complication rates have remained high (up to > 40%) [26–34]. The complexity of cPD is greater than that of PD. In addition, cPD yields more operative morbidities and mortalities [5–25], and PD performed during emergency conditions poses further risks [20, 27–31, 33]. The operative mortality of emergency PD exceeds 20 to 40% [20, 27–31, 33]. Patient preoperative conditions combined with the PD etiology are important contributors to surgical outcomes [29–31, 33].

Emergency resection of a bowel tumor is indicated for patients with acute abdomen caused by bowel obstruction, perforation or tumor bleeding [32].

Our hospital is the only government-supported tertiary referral medical center in central Taiwan. Patients often manifest life-threatening conditions related to colorectal carcinoma. Few studies have yet reported on emergency cPD for patients with locally advanced colorectal carcinoma presenting with serious acute abdominal conditions [30–32]. To this end, we retrospectively reviewed patients to evaluate the feasibility of emergency cPD for patients with locally advanced colorectal carcinoma presenting with an acute abdomen.

## Methods

Over a period of 25 years (from 1994 to 2018), we admitted 4898 patients for radical curative resection to treat their colorectal carcinoma. Of these patients, 30 had locally advanced colon carcinoma infiltrating the duodenum and/or pancreatic head that needed cPD. Of these 30 patients, 11 received cPD under emergency conditions (e-group). Their detailed information is shown in Table 1. The causes of emergency cPD in this group were as follows: 6 cases, acute bowel obstruction; 2 cases, tumor perforation following colonoscopic biopsy; 1 case, spontaneous tumor perforation; and 2 cases, tumor bleeding.

**Table 1:** Brief data of patients who underwent emergent colo-pancreaticoduodenectomy

Case no.	Sex	Age	Serum CEA Level (ng/mL)	Cause of emergency	Comorbidity	PD procedure	Complications	Hospital stay (days)	Operative blood loss (mL)	Total B.T (mL)	Present status
1	M	59	11.4	Iatrogenic perforation		PPPD	Wound infection, POPF (B)	28	1100	1000	DOD, 16 m
2	M	59	2.3	Iatrogenic perforation		PD	DGE (B)	33	550	0	NED, 34 m
3	F	66	1.7	Tumor bleeding	Diabetes mellitus	PPPD	DGE (A) BPC	27	600	500	DOD, 14 m
4	M	50	61.2	Bowel obstruction		PD		19	1000	2100	DOD, 52 m
5	F	44	369	Bowel obstruction		PD		22	800	2600	DOD, 27 m
6	F	36	1.0	Spontaneous perforation	Lupus erythematosus	PPPD		11	500	0	NED, 68 m
7	F	50	63.4	Bowel obstruction		PD	Bowel abscess, DGE (A)	74	600	0	DOOD, 54 m
8	M	43	1.6	Bowel obstruction		PD		8	500	0	NED, 120 m
9	M	52	19.8	Bowel obstruction		PPPD	Biliary leak (A) abscess	13	600	0	NED, 49 m
10	M	52	14	Bowel obstruction		PPPD	BPL Wound infection	16	500	0	DOD, 46 m
11	M	53	19	Tumor bleeding		PPPD	Wound infection	16	0	0	DOD, 52 m

Note: *CEA* carcinoembryonic antigen, *PPPD* pylorus-preserving pancreaticoduodenectomy, *NED* no evidence of disease, *DOD* died of disease, *DOOD* died of other disease, *DGE* delayed gastric empting time, *POPF* postoperative pancreatic fistula, *BPL* biochemical pancreatic leakage

During the same period, a total of 742 PDs were performed in our hospital. Patients who received cPD not due to locally advanced colorectal carcinoma or for other diseases were excluded from our study. Patients with locally advanced tumors extending to the duodenal wall with a well-preserved ampullary area but no need for cPD [9] were also excluded. The choice of classical PD or pylorus-preserving PD [26] was made based on the subjective evaluation of a gross negative margin present at the duodenal wall.

A senior hepato-biliopancreatic surgeon (CCW) performed or guided all 742 PDs during the study period. All perioperative assessments and operative procedures of cPD were performed by two senior surgeons (CCW and FKP), while colon cancer resection strategies were determined by another two colorectal surgeons (JBC and CCC), and two oncologists (CHC and YY) designed chemotherapies and target therapies.

Preoperative assessments for elective cPD, i.e., functional examinations of the lung, heart, liver, and kidney, were performed on all 19 patients in the non-e-group. Their comorbidities were well controlled. The level of carcinoembryonic antigen was measured. Computed tomography (CT) or magnetic resonance imaging was also performed.

Selection criteria for emergency cPD patients with obstruction and perforation were as follows: (1) age ≤ 60 years, (2) body mass index < 35 kg/m^2^, (3) no poorly controlled comorbidities in perforation cases, with an estimated perforated duration of ≤ 6 h with no severe intra-abdominal contamination. For the other 19 patients (non-e-group), after well-examined preoperative studies, their cPD procedure was performed under an elective regular schedule.

The preoperative assessments of patients with locally advanced colon cancer presenting with an acute abdomen were performed similarly for elective surgical treatments. These procedures were completed within 2 h following admission to the emergency department. Emergency laparotomy was carried out to alleviate acute abdominal conditions after fluid resuscitation and systemic antibiotic treatments. Diagnosis of the invasion severity of the colon cancer was typically made after exploratory laparotomy.

Because the general conditions of the patients did not meet the criteria outlined above, two patients with initially diagnosed perforation cases and one patient with obstruction were treated via diverting ileostomy with an omental patch to occlude perforation holes. Another, older patient with tumor obstruction was treated first by diverting ileostomy. The cPD procedure was then performed for these three patients 3 months later. They were categorized to the non-e-group.

Pancreaticojejunostomy is the key procedure for reconstructing pancreatic remnants and the gastrointestinal tract. In four non-e-group patients, the procedure involved the invagination method during the early periods of this study (prior to March 1996). For the later, 15 patients in the non-e-group and all patients in the e-group, 3 months after cPD, we performed staged pancreaticojejunostomy [30, 35, 36]. Regarding the management of colon-related complications, colon leakage was treated with diverting ileostomy, and intra-abdominal abscess was treated with percutaneous drainage (by surgeons JBC and CCC). Any complication or death occurring within 90 days after cPD was recorded as a surgical complication or as mortality. Complication severity was graded using the Dindo-Clavien classification [37]. Definitions and severities of pancreatectomy-related complications followed those of the International Study Group of Pancreatic Surgery. These complications included postoperative pancreatic fistula [38], postpancreatectomy hemorrhage [39], delayed gastric emptying [40], and bile leakage [41] and were graded based on the severity of pancreatectomy-related morbidities. After patients had recovered from cPD, they were followed-up monthly at the outpatient clinic during the next year and thereafter at intervals of 3–6 months. At each visit, CT or magnetic resonance imaging was performed, and serum levels of carcinoembryonic antigen were measured.

Chemotherapy or targeted therapy was routinely administered after cPD for a minimum of 2 years. All patients were followed-up until July 2020.

Resected specimens were sent to the pathology department to determine the pathological characteristics and cancer stages of colorectal carcinoma. For cancer cell differentiation, the definition by the World Health Organization [42] was used. For cancer staging, we used the benchmark classification of the American Joint Committee for Cancer (8th edition) [42]. The clinicopathological characteristics of patients and their early postoperative and long-term results after cPD were compared between the two groups.

### Statistical analysis

Continuous variables are presented as medians (ranges) and were compared using the Mann-Whitney *U* test. Frequencies were compared using Fisher’s exact test or Pearson’s *χ*^2^ test as appropriate. Rates of overall survival (OS) up to July 2020 were calculated using the Kaplan-Meier life table method and compared across groups using the log-rank test. Differences with *p* values < 0.05 were regarded as statistically significant.

## Results

All cPD procedures were performed by open laparotomy.

Table 2 shows the clinicopathological characteristics of the patients in both groups. Patient comorbidities in the non-e-group were old stroke in 2 cases, rheumatoid arthritis in 1 case, coronary artery disease in 2 cases, hypertension in 3 cases, obstructive lung disease in 2 cases, diabetes mellitus in 3 cases, end-stage renal disease in 1 case, and chronic hepatitis in 1 case (4 patients had ≥2 comorbidities).

**Table 2:** The clinico-pathological characteristics of patients who underwent colectomy and pancreaticoduodenectomy

Clinical characteristics	E-group (*n* = 11)	Non-e-group (*n* = 19)	*p*
Sex			
Male	7	8	0.449
Female	4	11	
Age (years)	52 (36.1–66)	70 (46–86)	< 0.001
Associated comorbidities	2	12	0.039
Serum carcinoembryonic antigen level (ng/mL)	11.4 (1.0–369.0)	8.0 (1.5–3498.0)	0.726
Body mass index (kg/m^2^)	23 (20.5–31)	22.5 (19.6–30.5)	0.776
Tumor characteristics			
Tumor size (cm)	7.5 (3.0–11.0)	7.5 (4.3–16.0)	0.331
Cancer differentiation			
Moderately differentiated	4	9	0.631
Poorly differentiated	7	10	
Depth of cancer invasion			
T4a (serosa)	3	2	0.327
T4b (adjacent organ)	8	17	
Lymph node metastasis	9	10	0.671
Dissected lymph node number	19 (13–46)	24 (15–69)	0.294
Radicality			
R0	9	18	0.587
R1	2	1	

Table 3 shows the early postoperative results after cPD. No patients succumbed from cPD in either group, yet complication rates appeared high in both groups, and there were no intergroup differences (64% in the e-group vs. 42% in the non-e-group, *p* = 0.449).

**Table 3:** Early postoperative outcomes after colectomy and pancreaticoduodenectomy

Early outcomes	E-group (*n* = 11)	Non-e-group (*n* = 19)	*p*
PD type			
Classical PD	6	9	0.867
Pylorus-preserving pancreaticoduodenectomy	7	7	
Operative time (hr)	8.0 (5.5–10.9)	7.5 (6.2–11.8)	0.746
Operative blood loss (mL)	600 (400–1100)	420 (150–3130)	0.268
Blood transfusion (mL)	0 (0–2600)	0 (0–2250)	0.955
Need for blood transfusion (No)	4	6	0.677
Complication	7 (63.6%)	8 (42.1%)	0.449
Wound infection	3	1	
Intra-abdominal abscess	2	2	
Postoperative pancreatic fistula grade B + C	2	2	
Delayed gastric empting	3	3	
Postpancreatectomy hemorrhage	0	1	
Grade B + C	0	1	
Dindo-Clavien severity grade 3 + 4	1	4	
Postoperative hospital day (days)	23 (18–74)	19 (9–45)	0.331
90-day mortality	0	0	1.000

One male patient in the non-e-group had dehiscence of an invaginated pancreaticojejunostomy (grade C postoperative pancreatic fistula) with gastroduodenal artery stump bleeding (grade C postpancreatectomy hemorrhage). It was rescued by emergency total separation and closure of both pancreatic and intestinal stumps, peripancreatic irrigation, and total parenteral nutrition [43, 44]. Half a year later, he developed a chronic pancreatic fistula that was treated with fistulojejunostomy [45].

Although all cPD procedures were grossly curative, pathological examination showed cancer exposure at the retroperitoneal dissection surface of the pancreatic head in three patients (1 in the non-e-group, 2 in the e-group). These cases were categorized as R1 resection, and the patients survived < 2.5 years.

**Fig. 1:**
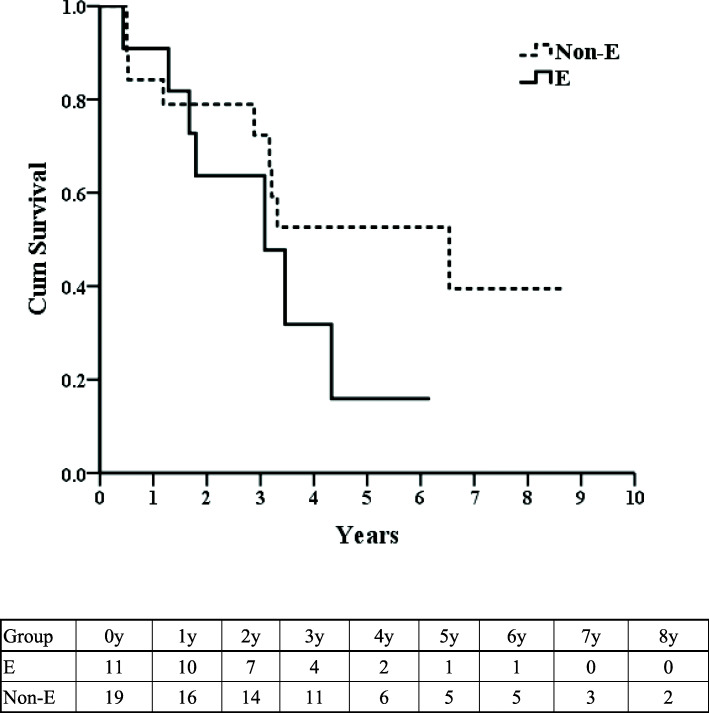
Overall survival rate after colo-pancreaticoduodenectomy

Figure 1 shows the OS rates of the two groups, with no intergroup differences (*p* = 0.192).

## Discussion

We reviewed our 25-year experience with cPD in patients with locally advanced colon cancer. A nationwide survey in the Netherlands reported that the most common indication of cPD is locally advanced pancreatic head cancer [23] that directly invades the colon or mesocolon, followed by locally invaded colon cancer at the duodenum and/or pancreatic head. The cPD procedure is rarely performed in gastrointestinal surgery. This is due to its complexity, difficulty, and high risks. In certain acute situations, cPD is the efficacious path forward. According to surgeons at the Memorial Sloan Kettering Cancer Center, New York, the most complex surgical procedures in cancer surgery are esophagectomy, hepatectomy, pancreatectomy, and total pelvic exenteration [44].

The above procedures are recommended as best performed at well-experienced medical centers. From our present study, we found similar postoperative courses for both cPD and PD. Therefore, successful emergency cPD may be similar to successful emergency PD. Emergency PD is associated with high postoperative morbidity and mortality, reaching 30% and 50% [30–33].

Gulle et al. [29] reported their operation as applied to 10 patients with emergency PD to treat complex pancreaticoduodenal trauma with zero deaths. However, their complication rate was high (80%). All their patients were relatively young and healthy without challenging comorbidities. Emergency PD for non-trauma cases has higher risks than that for trauma cases because of the often unrecognized preoperative poor conditions and coexisting inflammation or organ dysfunction that lead to failed emergency PD [30, 31, 33].

Managing postoperative complications after emergency cPD is also an important issue after emergency PD. Performing cPD is itself challenging and involves high skill levels and long operation times. It requires meticulous and experienced care during the preoperative evaluation period to minimize complications and deaths. Thus, the diagnosis and evaluation of preoperative general conditions of these patients should be well surveyed to prevent occurrences of potential postoperative adverse events. Therefore, proper preoperative selection of patients is critical for the success of cPD. Despite the high complication rates of emergency cPD in our patients, their rates of early and long-term survival appear acceptable.

Abdominal CT scans play an important role in the preoperative diagnosis of such advanced cancer. Attachment of colon tumor to duodenum and/or pancreas may lead us a suspicion of the disease. However, in patients who present with acute abdomen, CT scans facilitate the visualization of tumors due to marked intestinal dilatation or marked intra-peritoneal free air. When right colon cancer loosely adheres to the duodenal wall, it may be regarded as a duodenal invasion. Such cases were observed in our current study. Likewise, when a right colon cancer directly invades the pancreatic head, with a relatively small invaded area, the condition may be regarded as “no invasion”. Therefore, a definite diagnosis of colon cancer with duodenal or pancreatic invasion may be confirmed only after exploratory laparotomy. Moreover, damage-controlled procedures can be performed in patients not fulfilling our criteria for emergency cPD.

Patients with advanced age, poorly controlled comorbidities, unstable vital signs, or obesity are typically at risk of PD. They were therefore excluded from our emergency cPD procedure when treating bowel obstruction or perforation. Moreover, if the perforation time is long (> 6 h), severe intra-abdominal contamination could lead to edematous and fragile conditions. Long periods of generalized peritonitis may destroy sutures in the early postoperative period of cPD. These patients are therefore not recommended for emergency cPD.

Acute massive bleeding from gastrointestinal malignancy is very rare, but the sequelae are grave. Once it occurs, emergency resection to stop the bleeding is most likely the only life-saving option. The aforementioned patient selection criteria and management of obstruction and perforation are not applicable in bleeding cases. Trans-arterial embolization may be temporarily helpful for hemostasis [29, 46]. However, due to abundant vascular collaterals in pancreaticoduodenal regions, total hemostasis is difficult. The resection of a bleeding tumor resection is still mandatory after embolic control of hemostasis.

Tsai et al. reported that in emergency PD, intra-peritoneal infections have outcomes worse than bleeding [33]. In our series, we recommended 2-stage pancreaticojejunostomy after cPD.

Pancreaticojejunostomy has been considered the “Achilles tendon” of PD. For successful cPD, the management of the anastomosis is also crucial. During the early period of our study (before March 1996), we had a case of grade C catastrophic pancreatic leakage with bleeding (disruption of pancreaticojejunostomy with massive internal bleeding). The patient fortunately survived after our timely and appropriate management.

Intra-operative management for cPD adverse events, whether related or not related to techniques, is of great importance to reduce chances of operative mortality.

Staged pancreaticojejunostomy was routinely used for all PD after cases when the pancreatic duct size was small (< 2 mm) or when the pancreatic parenchyma was soft or associated with large vessel resection or a controlled, troublesome comorbidity.

Staged pancreaticojejunostomy was first proposed by Japanese surgeons, Miyagawa and Makuuchi, in 1994 [39]. They covered the common hepatic proper hepatic artery and gastroduodenal artery stump using a sheet of pedicled greater omentum or liver falciform ligament [35, 36]. A thin plastic tube was then inserted into the main pancreatic duct to totally exteriorize the pancreatic juice. Pancreatic juice was fed into the intestinal lumen through another tube jejunostomy, with the seromusculature sutured with the posterior wall of the pancreatic stump. The anastomosis was performed 3 months later by inserting the aforementioned plastic tube into the neighboring jejunal lumen.

The pedicled falciform ligament of the liver, or greater omentum, is capable of covering the transected stump of the gastroduodenal artery (the most common site of postpancreatectomy hemorrhage after PD) [39]. This vessel can be protected from erosion by leaked pancreatic juice. In fact, no catastrophic complications occurred or even minor leakage occurred after staged pancreaticojejunostomy. This further guaranteed the safety of cPD.

The experience of treating acute necrotizing pancreatitis is also helpful for treating pancreatic leakage after PD [46]. Appropriate and timely management of complications after PD may improve healing and prevent operative death. The pancreatectomy procedure for disrupted pancreatic anastomosis [28] has a high death rate [28, 40]. It should therefore be avoided [43]. Even with the development of chronic pancreatic fistula, treatment by fistulojejunostomy can be effective without undesirable sequelae [45].

Delayed gastric emptying is also a problematic adverse event in both PD and cPD. The event is likely related to the destruction of the upper abdominal autonomic nerve plexus during lymphadenectomy. The condition often requires prolonged hospitalization, long-term nasogastric decompression and total parenteral nutritional support. These management protocols could cause other systemic problems, such as catheter sepsis, electrolyte imbalance, trace element deficiencies, aspiration pneumonia and hepatic dysfunction. Some of these can be fatal. To avoid such severe complications, efforts to preserve the upper abdominal vagus nerve and sympathetic nerve plexus could minimize delayed gastric emptying. Upper abdominal lymphadenectomy, which is typically carried out for periampullary or pancreatic cancer, is not mandatory for colon cancer patients.

Total removal of locally advanced colon cancer is essential to obtain microscopic R0 resection. In our experience, R1 resection for colon cancer (cancer cells viewed microscopically after the operation) was actually palliative, as no patient who underwent R1 resection had survived for more than 3 years.

The 5-year OS for locally advanced colorectal cancer is 51%. The reported 5-year OS rate after cPD for advanced colon cancer is 50 to 60% [26]. Our non-e-group patients showed a prognosis comparable to that in the literature [4–11]. Because of the high incidence of lymph node metastasis, the prognosis of e-group patients is often poor. Nevertheless, both groups had similar OS. Histological TNM staging, lymph involvement and cancer cell differentiation are prognostic factors [5–12, 14, 15, 17–21, 23–25]. The cancer conditions of our patients receiving cPD were similar to those in the literature regarding colon cancer patients. The development of new target agents or chemotherapeutic drugs is helpful to prolong survival.

Several limitations of the current study are as follows.

First, our comparison of the two groups was not flawless. Patient selection or the choice of emergency procedure was based on perceived comorbidities of patients by the operating team, which could be quite arbitrary. Nevertheless, no differences were found in other variables.

Second, this was a longitudinal observational cohort study. It was not a randomized controlled trial because of the small sample size. Colon cancer that involves the duodenum and/or pancreas is a distinctively unique presentation. During the 25-year duration of our study, diagnostic tools, surgical techniques, operative equipment, and peri/preoperative assessments advanced markedly. Thus, our initial case-selection criteria in the e-group might have been too conservative. For example, the patient age could have been extended, as the life expectancy of the general population has increased by 5 years over the course of this study period [47]. It is reasonable to assume that the safety range of emergency cPD could have also been extended.

Third, treatment strategies for locally advanced colon cancer were chosen by our experienced colorectal and hepatopancreaticobiliary surgeons as well as oncologists. Over the study period, some of the involved members of this research study retired or shifted to other projects. Although a senior surgeon (CCW) has led the treatment strategy of individual patients in a constant manner, there is an inherent discrepancy in the continuity of management for this complex disease. For example, adjuvant therapies after elective or emergency cPD may have changed across different postoperative courses.

In the current study, we found no patients experiencing ileocolostomy (another important bowel anastomosis in cPD) leakage. Most postoperative complications were related to ileostomy, pancreatic, or colonic leakage, which could be managed by diverting the proximal presence of ileostomy necrosis. Nevertheless, abscesses could be treated by percutaneous drainage. These complications should be diagnosed early and promptly treated.

Fourth, although staged pancreaticojejunostomy may improve the safety of cPD in patients, additional admissions and operations are needed for a complete pancreaticojejunostomy. Given new safe-guarded techniques for dealing with pancreaticoanastomosis, the safety of cPD in a single operation could be developed, reducing both hospital costs and anesthetic risks.

Despite high complication rates, our reviewed experiences support the conclusion that emergency cPD is a feasible procedure in highly selected patients with locally advanced colon cancer presenting acute abdomen. The long-term outcomes after emergency and nonemergency cPD are comparable.

## Conclusions

Emergency cPD is feasible in highly selected groups when performed by only experienced colorectal and hepato-biliopancreatic surgeons. The early and long-term positive outcomes of emergency cPD are similar to those after nonemergency cPD in patients with life-threatening acute abdominal conditions.

## Data Availability

The datasets used or analyzed during the current study are available from the corresponding author on reasonable request.

## Abbreviations

PD: Pancreaticoduodenectomy
cPD: Colo-pancreaticoduodenectomy
CT: Computed tomography
OS: Overall survival
